# Supplementary material for: Spinal muscular atrophy genetic epidemiology and the case for premarital genomic screening in Arab populations
Source: Commun Med (Lond). 2024 Jun 15;4:119. doi: 10.1038/s43856-024-00548-1 (PMC11180197; doi:10.1038/s43856-024-00548-1)
Supplement: Supplementary file 2 — Description of Additional Supplementary Files [file 43856_2024_548_MOESM2_ESM.pdf]

Description of Additional Supplementary Files

File name: Supplementary Data 1

File Description: Source data file.
