# Supplementary material for: Spinal muscular atrophy genetic epidemiology and the case for premarital genomic screening in Arab populations
Source: Commun Med (Lond). 2024 Jun 15;4:119. doi: 10.1038/s43856-024-00548-1 (PMC11180197; doi:10.1038/s43856-024-00548-1)
Supplement: Supplementary file 4 — Reporting Summary [file 43856_2024_548_MOESM4_ESM.pdf]

Corresponding author(s): DAPR COMMSMED-24-0040

Last updated by author(s): Mar 18, 2024

Reporting Summary

Nature Portfolio wishes to improve the reproducibility of the work that we publish. This form provides structure for consistency and transparency in reporting. For further information on Nature Portfolio policies, see our Editorial Policies and the Editorial Policy Checklist.

Statistics

For all statistical analyses, confirm that the following items are present in the figure legend, table legend, main text, or Methods section.

n/a	Confirmed	
☐	☒	The exact sample size (*n*) for each experimental group/condition, given as a discrete number and unit of measurement
☒	☐	A statement on whether measurements were taken from distinct samples or whether the same sample was measured repeatedly
☐	☒	The statistical test(s) used AND whether they are one- or two-sided<br>*Only common tests should be described solely by name; describe more complex techniques in the Methods section.*
☒	☐	A description of all covariates tested
☒	☐	A description of any assumptions or corrections, such as tests of normality and adjustment for multiple comparisons
☒	☐	A full description of the statistical parameters including central tendency (e.g. means) or other basic estimates (e.g. regression coefficient) AND variation (e.g. standard deviation) or associated estimates of uncertainty (e.g. confidence intervals)
☒	☐	For null hypothesis testing, the test statistic (e.g. F, t, r) with confidence intervals, effect sizes, degrees of freedom and P value noted<br>*Give P values as exact values whenever suitable.*
☒	☐	For Bayesian analysis, information on the choice of priors and Markov chain Monte Carlo settings
☒	☐	For hierarchical and complex designs, identification of the appropriate level for tests and full reporting of outcomes
☒	☐	Estimates of effect sizes (e.g. Cohen's *d*, Pearson's *r*), indicating how they were calculated

Our web collection on statistics for biologists contains articles on many of the points above.

Software and code

Policy information about availability of computer code

Data collection	Not applicable
Data analysis	Not applicable

For manuscripts utilizing custom algorithms or software that are central to the research but not yet described in published literature, software must be made available to editors and reviewers. We strongly encourage code deposition in a community repository (e.g. GitHub). See the Nature Portfolio guidelines for submitting code & software for further information.

Data

Policy information about availability of data

All manuscripts must include a data availability statement. This statement should provide the following information, where applicable:

- Accession codes, unique identifiers, or web links for publicly available datasets
- A description of any restrictions on data availability
- For clinical datasets or third party data, please ensure that the statement adheres to our policy

All data is included in the manuscript

Research involving human participants, their data, or biological material

Policy information about studies with human participants or human data. See also policy information about sex, gender (identity/presentation), and sexual orientation and race, ethnicity and racism.

Reporting on sex and gender	Sex distribution in clinical cohort and screening cohort were almost equally distributed, having 51% males and 49% females in both.
Reporting on race, ethnicity, or other socially relevant groupings	Clinical cohort consisted mostly of Arab and Middle Eastern pediatric patients referred for clinical testing. Screening cohort consisted of local Emirati newborns from different parts across the country.
Population characteristics	See above
Recruitment	Parents/ Guardians of newborns were randomly approached and consented to participate (written consent) in the study.
Ethics oversight	The de-identified reporting of clinical cohort in this study was approved by the Dubai Healthcare Authority Research Ethics Committee (DSREC-07/203_06), which determined that this study meets the exemption criteria with a waiver of informed consent since all data were de-identified.<br><br>The study was approved by the Dubai Health Authority Scientific Research Ethics Committee (DSREC-06/2021_20), Ministry of Health and Prevention Research Ethics Committee (MOHAP/DXB-REC/SOO/No.81/2021) and Abu Dhabi Health Research and Technology Ethics Committee (DOH/CVDC/2022/1626). Parents/guardians of all recruited newborns provided written informed consent.

Note that full information on the approval of the study protocol must also be provided in the manuscript.

Field-specific reporting

Please select the one below that is the best fit for your research. If you are not sure, read the appropriate sections before making your selection.

☒ Life sciences ☐ Behavioural & social sciences ☐ Ecological, evolutionary & environmental sciences

For a reference copy of the document with all sections, see nature.com/documents/nr-reporting-summary-flat.pdf

Life sciences study design

All studies must disclose on these points even when the disclosure is negative.

Sample size	SMA incidence in other populations can be as low as 1 in 7000. In Middle East it's expected to be lower due to consanguinity. In addition, our study relies on allele frequency (and therefore carrier rate) to derive incidence. Therefore a sample size around >1000 was considered adequate
Data exclusions	No data were excluded from the study
Replication	Not applicable
Randomization	Emirati newborns were randomly recruited
Blinding	Not applicable

Reporting for specific materials, systems and methods

We require information from authors about some types of materials, experimental systems and methods used in many studies. Here, indicate whether each material, system or method listed is relevant to your study. If you are not sure if a list item applies to your research, read the appropriate section before selecting a response.

Materials & experimental systems

n/a	Involved in the study
☒	☐ Antibodies
☒	☐ Eukaryotic cell lines
☒	☐ Palaeontology and archaeology
☒	☐ Animals and other organisms
☒	☐ Clinical data
☒	☐ Dual use research of concern
☒	☐ Plants

Methods

n/a	Involved in the study
☒	☐ ChIP-seq
☒	☐ Flow cytometry
☒	☐ MRI-based neuroimaging

Plants

Seed stocks	NA
Novel plant genotypes	NA
Authentication	NA
